# DeepBrainSeg: Automated Brain Region Segmentation for Micro-Optical Images With a Convolutional Neural Network

**DOI:** 10.3389/fnins.2020.00179

**Published:** 2020-03-20

**Authors:** Chaozhen Tan, Yue Guan, Zhao Feng, Hong Ni, Zoutao Zhang, Zhiguang Wang, Xiangning Li, Jing Yuan, Hui Gong, Qingming Luo, Anan Li

**Affiliations:** ^1^Britton Chance Center for Biomedical Photonics, Wuhan National Laboratory for Optoelectronics, Huazhong University of Science and Technology, Wuhan, China; ^2^MoE Key Laboratory for Biomedical Photonics, School of Engineering Sciences, Huazhong University of Science and Technology, Wuhan, China; ^3^HUST-Suzhou Institute for Brainsmatics, JITRI Institute for Brainsmatics, Suzhou, China

**Keywords:** automated segmentation, brain regions, convolutional neural networks, image registration, domain-condition constraints, micro-optical images

## Abstract

The segmentation of brain region contours in three dimensions is critical for the analysis of different brain structures, and advanced approaches are emerging continuously within the field of neurosciences. With the development of high-resolution micro-optical imaging, whole-brain images can be acquired at the cellular level. However, brain regions in microscopic images are aggregated by discrete neurons with blurry boundaries, the complex and variable features of brain regions make it challenging to accurately segment brain regions. Manual segmentation is a reliable method, but is unrealistic to apply on a large scale. Here, we propose an automated brain region segmentation framework, DeepBrainSeg, which is inspired by the principle of manual segmentation. DeepBrainSeg incorporates three feature levels to learn local and contextual features in different receptive fields through a dual-pathway convolutional neural network (CNN), and to provide global features of localization by image registration and domain-condition constraints. Validated on biological datasets, DeepBrainSeg can not only effectively segment brain-wide regions with high accuracy (Dice ratio > 0.9), but can also be applied to various types of datasets and to datasets with noises. It has the potential to automatically locate information in the brain space on the large scale.

## Introduction

Complex structures in the brain have the specificity for brain regions, which correspond to varying brain functions. The maturation of techniques for high-resolution micro-optical imaging (Li et al., 2010; Ragan et al., 2012; Gong et al., 2016) has allowed comprehensive measurements of the distributions of fine structures in three-dimensional (3D) brain space. This has led to better understanding of brain structures, such as whole-brain neuron projections (Economo et al., 2016; Li et al., 2018), cellular and vascular distributions (Peng et al., 2017; Xiong et al., 2017). Such analyses require 3D brain region contours as boundary preconditions. However, unlike magnetic resonance images (MRIs), brain regions in microscopic images are aggregated by discrete neurons, resulting in blurry boundaries between regions (Gahr, 1997). Identifying the boundaries requires to combine a number of features, including cellular staining, morphology, and distribution. Moreover, due to individual differences and imaging processes, these complex features are variable, making it challenging to accurately segment brain regions. The manual segmentation of brain region contours (Dong, 2008) by anatomists is considered to be a reliable method, but is unrealistic to apply on a large scale for high-resolution images. Therefore, neuroscientists urgently require an automated and accurate method that can segment brain regions at the cellular level.

Image segmentation has been studied extensively for brain sciences. Classic segmentation methods (Clarke et al., 1995; Balafar et al., 2010; Nanthagopal and Sukanesh, 2013) based on hand-crafted features have been used for a long time and primarily utilize the differences between features, such as intensity and texture. For example, Feng et al. (2017) used a 3D Otsu method with intensity features to segment MRI brain structures. However, the features of brain regions for micro-optical images are complex, and vary between different individuals and imaging devices, rendering the hand-crafted features approach inappropriate for micro-optical brain images.

Deep learning (LeCun et al., 2015; Schmidhuber, 2015; Shen et al., 2017) for image segmentation is another rapidly developing field. Methods based on convolutional neural networks (CNNs) (Krizhevsky et al., 2012; Rawat and Wang, 2017) can build complex deep-level features based on simple low-level features, making them competitive against classic shallow hand-crafted features approaches. One approach for image segmentation which uses CNNs has an end-to-end form with full convolutions (Long et al., 2015; Milletari et al., 2016; Badrinarayanan et al., 2017; Chen et al., 2017; Jégou et al., 2017; Yu et al., 2017; Chen et al., 2018); i.e., the output of the network is the result of pixel-by-pixel segmentation. For instance, U-net (Ronneberger et al., 2015), consisting of groups of convolutional and deconvolutional layers and skip links, is widely applied in medical image segmentation. Whereas, due to pooling layers, the end-to-end approach may adversely affect the image resolution and therefore result in loss of details (Litjens et al., 2017). Moreover, since a whole image constitutes one sample, many hours of labor are required to label enough samples for training.

Another CNN approach, the patch-based method (Lai, 2015; Pereira et al., 2016), is able to handle the details and label samples to an acceptable level. This approach classifies each pixel in the image individually by presenting it with patches extracted around that particular pixel (Litjens et al., 2017). For example, Ciresan et al. (2012) used a patch-based CNN to segment medical images; furthermore, multi-scale CNNs (de Brebisson and Montana, 2015; Moeskops et al., 2016) were adopted to achieve a higher accuracy for MR brain images with different receptive fields (Luo et al., 2016). However, the patch-based approach has the limitations of low efficiency and lack of global information.

Neuroanatomical studies benefit from the ability to obtain high-resolution micro-optical images, which allows fine division of the brain into thousands of regions (Kuan et al., 2015). The steps for manual segmentation of brain regions by anatomists consist in locating the structure at the macroscale, identifying the shape and neighboring differences at the mesoscale, and segmenting accurate boundaries at the microscale. Correspondingly, the automated segmentation also requires multi-level features: global, contextual, and local. While CNN methods can learn local and contextual features, they have difficulty utilizing global location features from the whole-brain range at high resolution, resulting in over-segmentation for other regions with similar local features. To locate brain structures, Iqbal et al. (2019) segmented and classified the mouse brain into eight regions using Mask r-cnn (He et al., 2017), while the detected box has excessive redundancies for the region with complex shape. Chen et al. (2019) combined a patch-based CNN and registration to segment the murine brainstem, whereas the accuracy of segmentation is easily affected by the effect of registration. In other words, current automated methods are not capable of utilizing on global, contextual, and local information to accurately segment brain regions for micro-optical images.

We propose a framework inspired by the principle of manual segmentation, DeepBrainSeg, which automatically locates and segments brain regions incorporating three level features: local, contextual, and global. We design a dual-pathway network with two-scale patches to acquire local and contextual features in different receptive fields, and combine image registration and domain-condition constraints for initial and tracking localization. We segmented several brain-wide regions and quantitatively evaluated the segmentation effect: which shows a high accuracy (Dice ratio > 0.9). DeepBrainSeg achieves more accurate results than U-net, V-net, FC-DensNet, and Segnet. It is also suitable for datasets with noises and can be used for various types of datasets. In addition, DeepBrainSeg demonstrates high computational efficiency on different platforms.

## Materials and Methods

### Biological Datasets

In this study, we used 14 mouse brain datasets from four different imaging systems. Ten datasets are Thy1-GFP M-line transgenic mice whose whole brains are imaged using a dual color fluorescence microscope [Brain-wide Precision Imaging system (BPS)] (Gong et al., 2016). The other four datasets are a Nissl-stained C57BL/6 adult mouse imaged using a Micro-Optical Sectioning Tomography (MOST) system (Li et al., 2010), a C57BL/6 mouse with autofluorescent signal imaged with a serial two-photon (STP) system (Ragan et al., 2012), a C57BL/6 adult mouse imaged with MR image model T2^∗^ (Johnson et al., 2010), and the Allen mouse common coordinate framework (Allen CCF v3 brain atlas) containing an 3D average brain image and a labeled brain region space. We got the STP dataset from “http://www.swc.ucl.ac.uk/aMAP,” the MR dataset from “civmvoxport.vm.duke.edu,” and the Allen CCF from “https://atlas.brain-map.org.” The pixel resolution of the MR dataset is 21.5 μm isotropic; others are all sampled to 10 μm isotropic.

### The Framework of DeepBrainSeg

**FIGURE 1:**
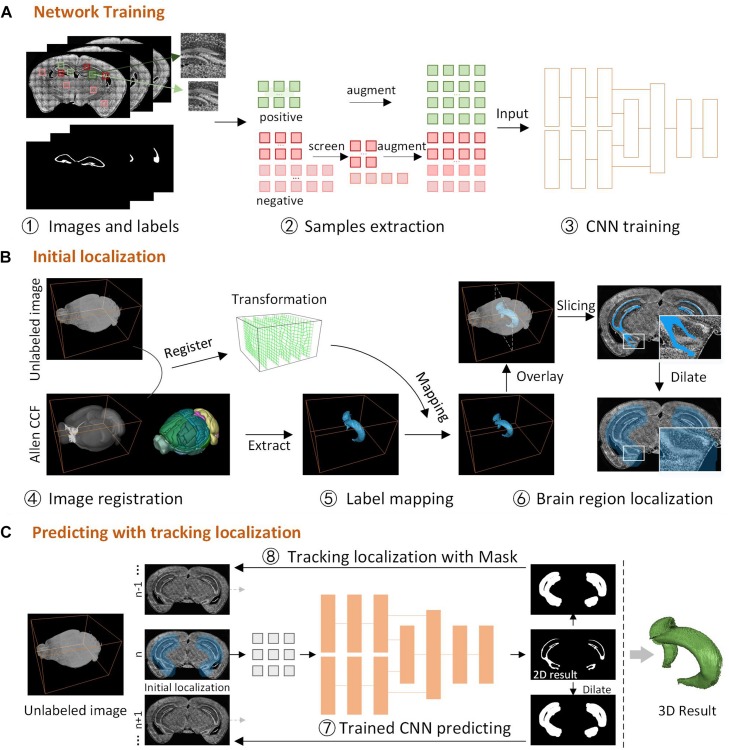
The framework for DeepBrainSeg. **(A)** Network training, the acquisition of images and labels, samples extraction, building and training the CNN. **(B)** Initial localization, image registration for the unlabeled image and Allen CCF, mapping the label to the image, and initial localization the brain region. **(C)** Predicting with tracking localization, predicting the initial image, dilating the 2D result as the localization of adjacent images, alternating prediction and localization to obtain a 3D result.

DeepBrainSeg consists of three parts (Figure 1): network training, initial localization, and predicting with tracking localization. First, we obtain images and labels by manually delineating the boundaries of brain regions, screen and augment the samples to generate the training set, and train the designed dual-pathway CNN (Figure 1A). Then, for the new unlabeled image, we perform a 3D registration with Allen CCF, and map the label from the Allen CCF to the unlabeled image, select one two-dimensional (2D) label slice and dilate it as the initial localization of the brain region (Figure 1B). Finally, the located 2D image is used for predicting by the trained CNN, and the segmentation result is dilated as the domain-condition constraint to locate the adjacent images. Tracking localization and prediction are performed alternately until the complete 3D segmentation results are obtained (Figure 1C).

### Label and Sample Extraction

The main datasets used for verification in this study are 10 datasets from BPS. Five brain regions with visible differences in the surrounding areas were selected for training and predicting: main olfactory bulb mitral layer (MOBmi), pyramidal and granular layers of the hippocampus (HIP-pg), the granular layer of cerebellar cortex (CBX-gl), outline, and facial nerve (VIIn). For each brain region, 100 coronal planes from five datasets were selected at intervals as the training and predicting images. Subsequently, using the Amira (version 6.1.1; FEI, Mérignac Cedex, France) tool, three experienced technicians generated the “labels” by manually demarcating the boundaries of the brain region on training and predicting images, to be used as the ground truth (Figure 1A.1).

For the dual-pathway CNN, a sample is presented as images with two different sizes around the particular pixel, and the value of the pixel in the label is the classification. There are two common problems in the sample extraction: there is much redundancy between adjacent patches, and the number of patches where the center pixel is within the brain regions (the positive samples) is much smaller than in other regions (the negative samples). To solve these problems, we customized the sample extraction scheme according to the characteristics of the brain regions (Figure 1A.2). First, we extract samples at intervals on coronal images to avoid excessive repetitive information. Then the data are screened and augmented (Krizhevsky et al., 2012) to maintain the equilibrium of positive and negative samples. The augmentation extends the intensity range in the data to improve the ability of the model for generalization. The process is as follows: randomly remove 90% of negative samples containing no pixel in the brain regions; randomly remove *x*% negative samples containing parts of pixels in the brain regions; augment the rest of samples by increasing and decreasing the intensity by 20%. The equilibrium of positive and negative samples is as follows:

(1) $$10\%\,N_{1}+3\cdot x\%\,N_{2}=3\,N_{3}$$

where N_1_ and N_2_ are the number of negative samples containing no pixel and parts of pixels in the brain regions, respectively, and N_3_ is the number of positive samples. Finally, we extracted hundreds of thousands of training samples for each brain region, of which 80% were used as the train set and 20% as the validation set.

### Dual-Pathway CNN Training

**FIGURE 2:**
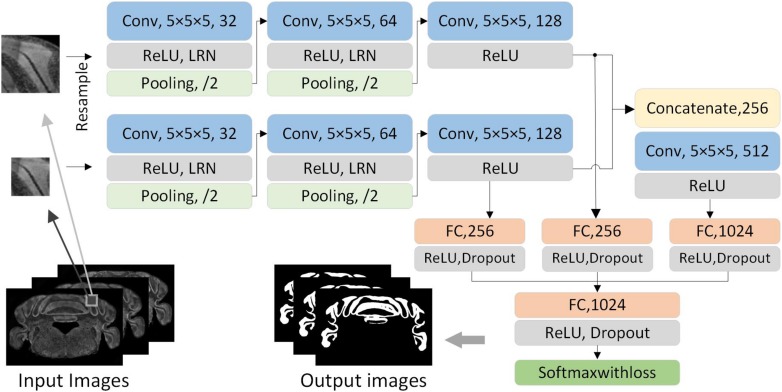
The architecture of dual-pathway CNN. The network consists of dual pathways that take the smaller and larger patch as input, respectively. Each pathway has three hidden layer which have the main components of a convolutional layer, a ReLU layer, an LRN layer, and a pooling layer. The dual-pathway feature maps form the input to a full connection and a convolution layer. All are concatenated after a full connection, and the SoftmaxWithLoss classifier is applied at the end.

In order to acquire the local and contextual features from different receptive fields, we designed a dual-pathway CNN with two-scale patches to segment brain regions. The smaller patches mainly provide local features while the larger patches provide contextual features. As shown in Figure 2, the network first consists of two same-pathway structures with three hidden layers. The first two hidden layers consist of a convolutional layer, an active layer, a local response normalization (LRN), and a pooling layer. The convolution kernel is 5 × 5, the stride is 1 × 1, the activation layer uses rectified linear units (ReLUs), the pooling layer uses 3 × 3 max-pooling, and the stride is 2 × 2. The third hidden layer consists of a convolutional layer and an active layer. The two-pathway network results in 128 5 × 5 feature maps. Subsequently, the feature maps are cascaded and connected by a 5 × 5 convolutional layer and a ReLU to acquire 512 1 × 1 feature maps. Then, the feature maps from the third and the fourth layer hidden layers are input to the corresponding fully connected layers. All the feature maps are concatenated and input to a fully connected layer. Following this, ReLU is applied, and dropout is used to prevent overfitting. Finally, the SoftmaxWithLoss classifier is used to handle the feature maps. The softmax function is defined as follows:

(2) $$\sigma_{\text{i}}\,\left(z\right)=\frac{\exp\left(z_{i}\right)}{\sum_{j=1}^{m}\exp\left(z_{j}\right)},i=1,\ldots ,m$$

The multinomial logistic loss function is defined as

(3) $$\ell \,\left(y,o\right)=-\log\left(o_{y}\right)$$

Finally, the combined softmax and loss function are expressed as

(4) $$\tilde{\ell }\left(y,z\right)=-\log\left(\frac{e^{z_{y}}}{\sum_{j=1}^{m}e^{z_{j}}}\right)=\log\left(\sum_{j=1}^{m}e^{z_{j}}\right)-z_{y}$$

The network training was implemented through Caffe (Jia et al., 2014) to obtain five models of the corresponding brain regions. During the training process, the batch size is 200, and the maximum number of iterations is 50,000 with 100 epochs. The learning rate is initialized to 0.01, and the iterative decay algorithm by step is applied every 10,000 iterations. The momentum and weight decay are 0.9 and 0.0005, respectively. The training is executed on the GPU to improve the efficiency.

### Initial Localization by Image Registration

It is necessary to locate brain regions before predicting the segmentation result to avoid over-segmentation and to improve efficiency. Brain atlas is commonly used as a reference for brain region recognition. Here, we use Allen CCF to locate the brain region by mapping the segmented labels to new images. Allen CCF consists of a 3D average brain image and a corresponding labeled brain region space. First, we register the unlabeled image and the average brain in 3D to obtain the transformation (Figure 1B.4). Then, the label for corresponding brain region from Allen CCF is extracted, and the label is mapped to the new image with the transformation (Figure 1B.5), which enables general localization of brain regions. However, due to differences in biological samples and imaging mode, it can be difficult to guarantee an accurate match between the mapped label and brain region, especially where brain regions appear and disappear. Instead of locating the whole 3D brain region, we select a 2D label from the middle slice of the 3D label as the initial localization and then perform a dilation of the label to eliminate registration errors, which ensures that all pixels within the brain region are included in the dilated label (Mask-init) (Figure 1B.6).

For image registration, a multi-resolution pyramid strategy is used for acceleration. Each hierarchy contains both linear and non-linear registration, and aims to maximize mutual information between the unlabeled image and the average brain. Symmetric diffeomorphic normalization (Avants et al., 2008), a widely used method, is conducted as the non-linear transformation model. Its energy function is defined as

(5) $$\begin{array}{c} E_{s\,y\,m}\,\left(I,J\right)=\underset{\phi_{1}}{\inf}\underset{\phi_{2}}{\inf}\int_{t=0}^{0.5}\left\{{||v_{1}\,\left(x,t\right)||}_{L}^{2}+{||v_{2}\,\left(x,t\right)||}_{L}^{2}\right\}\,dt+\\ \;\int_{\Omega }|I(\phi_{1}\left(0.5\right)-J(\phi_{2}\left(0.5\right){|}^{2}d\Omega \end{array}$$

where *v*_1_ and *v*_2_ are the velocity field in opposite directions and ∅_1_and ∅_2_ are the diffeomorphism field in opposite directions.

### Simultaneous Tracking Localization and Prediction

**FIGURE 3:**
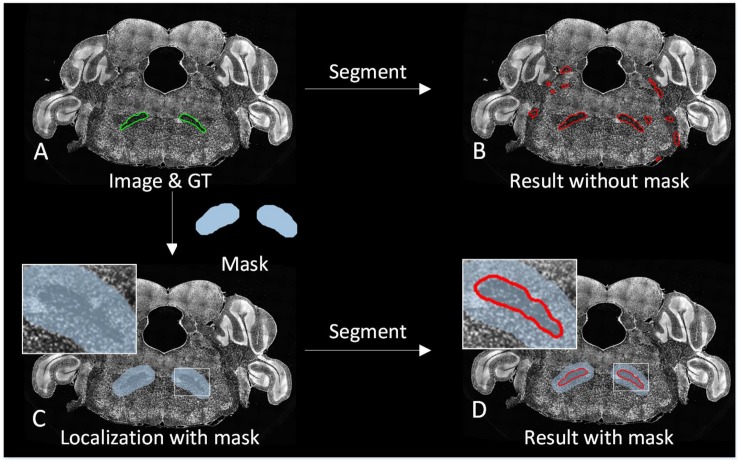
Comparison of the segmentation effect with and without localization. **(A)** A superposition of the original image and the manually segmented lines. **(B)** The segmentation result without localization. **(C)** A superposition of the original image and Mask. **(D)** The segmentation result with localization.

The 3D brain region can be regarded as changes of the 2D brain region slice in the spatial domain. High axial resolution imaging makes adjacent 2D brain region slice change less, making it possible to track the 2D brain region in a similar way to target tracking on a video in the time domain. Based on this idea, we proposed a strategy to locate the brain region during the prediction. For 3D brain region segmentation, initial localization by image registration is performed as the first predicting image with Mask-init, patches in the Mask-init are extracted from the 2D image as the input of trained CNN, and the 2D segmentation result is obtained through network predicting (Figure 1C.7). Subsequently, we dilate the 2D result as the domain-condition constraint (Mask-track) of the adjacent images (Figure 1C.8). For adjacent images, the network predicts patches in the Mask-track to get the 2D result. Finally, alternate tracking localization and prediction are performed for the rest of the corresponding 2D images to obtain a 3D segmentation result, and postprocessing operations including hole filling, connected component analysis, and 3D smoothing are conducted. Figure 3 demonstrates the segmentation effect with and without Mask-track, localization can avoid over-segmentation of similar local features.

In addition, only the pixels in the Mask-track require predicting, which greatly improves the efficiency. Moreover, based on the connected domain characteristics, brain regions are predicted at one pixel interval to reduce computation by three quarters. These optimizations make it efficient for high-resolution images.

### Quantitative Evaluation

To assess the accuracy of our method, we used three parameters to evaluate the segmentation effect: Dice (Dice, 1945), Precision, and Recall. The corresponding formulae are as follows:

(6) $$\text{Dice}\,\left(I,J\right)=\frac{2\times \left|I\cap J\right|}{\left|I\right|+\left|J\right|}$$

(7) $$\text{Precision}\,\left(I,J\right)=\frac{\left|I\cap J\right|}{\left|I\right|}$$

(8) $$\text{Recall}\,\left(I,J\right)=\frac{\left|I\cap J\right|}{\left|J\right|}$$

where I and J represent automated and manual binarized segmentation images, | I| and | J| denote the numbers of pixels in brain regions, and | I∩J| denotes the intersection of | I| and | J| for the pixels in brain regions.

In addition, we also quantitatively assess the effect of localization by Precision and Recall, where I represents automatically located binarized images (Mask-init and Mask-track).

### Testing Environments

We tested our method on two different computing platforms: A graphical workstation equipped with a NVIDIA M6000 GPU card, 20 CPU cores (Intel Xeon E5-2687w × 2), and 128 GB of RAM. A GPU server equipped with four NVIDIA V100 GPU cards, 12 CPU cores (Intel Xeon xeon-6126w × 2), and 192 GB of RAM.

## Results

### Determination of Dilation Sizes and Two-Scale Path Size

Here, we experimentally determined the optimal values for parameters by investigating different dilation sizes of Mask-init, Mask-track, and two-scale patch size. Since CBX-gl can be wide-ranging in terms of sizes, we used it as a representative for experiment using 10 BPS datasets. For testing of dilation size, Precision indicates the redundancy of localization range for the ground truth, and Recall indicates the accuracy of the localization. To ensure subsequent segmentation accuracy, Recall must be very close to 1. We therefore assessed sizes of 10, 20, 30, 40, and 50 pixels. As shown in Table 1, Recall ratio for Mask-init increases as the dilation size increases, it achieves the highest of 0.999 at 50 pixels, and Recall ratio for Mask-track reaches 0.999 at 20 pixels. Therefore, we determine the optimal dilation sizes for Mask-init and Mask-track with 50 pixel and 20 pixels, respectively.

**TABLE 1:** Performance of Recall ratio for Mask-init and Mask-track with different dilation size (bold values are the optimal).

Dilate size	10 pixels	20 pixels	30 pixels	40 pixels	50 pixels
Mask-init	0.807 ± 0.063	0.936 ± 0.032	0.975 ± 0.017	0.994 ± 0.005	**0.999 ± 0.001**
Mask-track	0.997 ± 0.008	**0.999 ± 0.005**	0.999 ± 0.004	1.000 ± 0.002	

**FIGURE 4:**
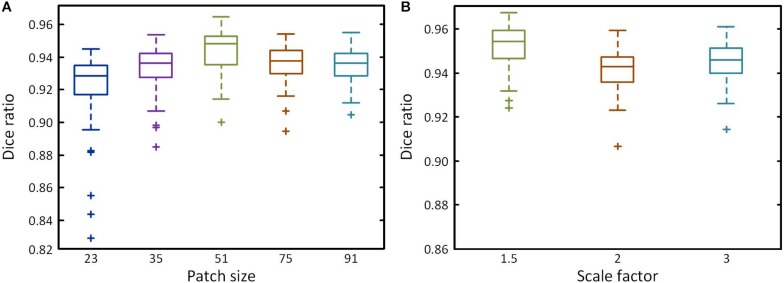
Performance of different patch size. **(A)** Box plots showing the Dice ratio for five different patch sizes at a single scale. **(B)** Box plots showing the Dice ratio for the larger patch at different multiples of the smaller patch size.

For two-scale path size, the receptive field will increase as patch size increases with the amount of information in a wide area. This improves classification accuracy, but also reduces positioning accuracy. To obtain optimal patch sizes, two groups of tests were conducted by first determining the smaller size patches and then the larger size using Dice ratio. First, five patch sizes (23, 35, 51, 75, and 91) were chosen to build single-scale networks. As the patch size increases, the wider receptive field improves classification accuracy, decreasing the numbers of outlier, and Dice ratio gradually increases (Figure 4A), reaching a peak at 51 pixels^2^ (Dice ratio = 0.944), after which it declines. We therefore selected 51 pixels^2^ as the optimal parameter for the smaller size. Then, the selected smaller patch size is multiplied by 1.5, 2, or 3 times to produce the larger patch sizes. Figure 4B shows the Dice ratio for different multiples. The highest value is obtained with a multiplication factor of 1.5 (Dice ratio = 0.952), after which it declines as the reduction of positioning accuracy has a major impact. Meanwhile, Dice ratio also reveals that the accuracy of two-scale is higher than the single-scale. We ultimately obtained the optimal patch sizes of 51 × 51 and 77 × 77 pixels^2^ for brain segmentation.

### Segmentation for Brain-Wide Regions

**FIGURE 5:**
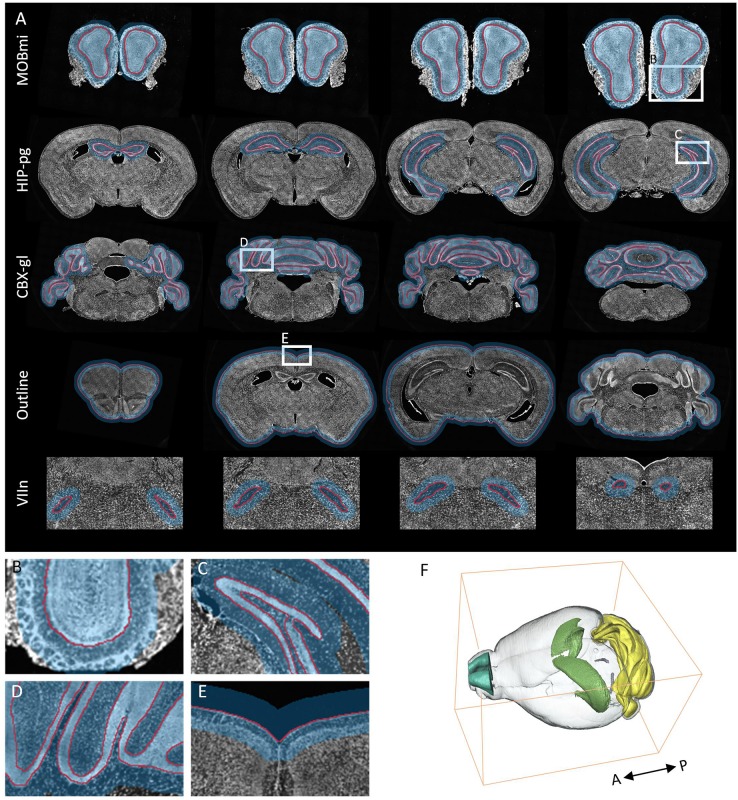
Segmentation effects for brain-wide regions. **(A)** The segmentation effects for five brain regions. From top to bottom: MOBmi, HIP-pg, CBX-gl, VIIn, and outline, each of which are shown as the superposition of four typical coronal images, localization masks, and segmented lines. **(B–E)** Enlarged views of the white boxes in **A**. **(F)** A 3D reconstruction of the segmentation results of the five brain regions.

In the field of neuroscience, the analysis of brain space and information commonly requires the segmentation of multiple brain regions which are distributed throughout the brain. Here, we selected five brain regions from ten BPS datasets for segmentation (see section “Materials and Methods” for specific training and prediction procedures). Using the trained models for each brain region, we performed localization and prediction for 50 corresponding images from five datasets. One dataset is used to illustrate the effects of localization and segmentation, by showing the overlapping of the original images, the located Mask, and the segmented lines from the binarized results (Figure 5). Figure 5A reveals the overall effects for the five brain regions (MOBmi, HIP-pg, CBX-gl, VIIn, and outline). Although there are differences in the characteristics among each brain region, DeepBrainSeg displays good localization and segmentation effects on all of these regions. Figures 5B–E show enlarged images of the white boxes in Figure 5A. The segmented lines are close to the real boundaries in the detail images. In particular, HIP-pg and CBX-gl, which have complicated shapes, also maintain fine effects. Figure 5F shows a 3D reconstruction of the segmentation results, which demonstrates the integrity and continuity of our approach in 3D space.

**FIGURE 6:**
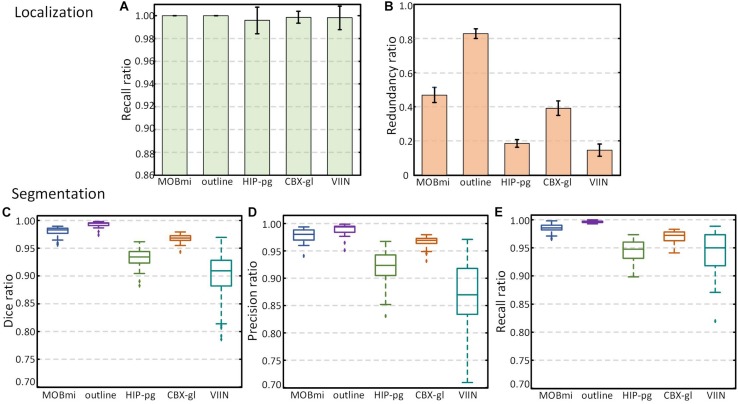
Performance of DeepBrainSeg for brain-wide regions. **(A,B)** Recall and Redundancy of localization effect. **(C–E)** Box plots showing Dice, Precision, and Recall (from left to right) of segmentation effect.

We also quantitatively evaluated the performance of localization and segmentation for these 50 images from five brain regions. Figures 6A,B show Recall and Precision (Redundancy) ratio for localization. Recall of all brain regions is very close to 1, indicating that almost all pixels of brain regions are included in the Mask, and Redundancy is between 0.14 and 0.83 for different regions. Figures 6C–E demonstrate box plots of Dice, Precision, and Recall for the segmentation effect. All three parameters exceed 0.95 for MOBmi, CBX-gl, and outline, and 0.92 for the complex HIP-pg structure. Although subtle deviations in the automated segmentation will affect the parameters for small brain regions, the parameters are consistently above 0.85 for VIIn. Detailed performance statistics showing means and standard deviations are provided in Table 2.

**TABLE 2:** Performance of DeepBrainSeg for brain-wide regions.

		MOBmi	Outline	HIP	CB	VIIn
Localization	Recall	1.000 ± 0.000	1.000 ± 0.000	0.996 ± 0.012	0.999 ± 0.005	0.998 ± 0.010
	Redundancy	0.468 ± 0.044	0.828 ± 0.028	0.186 ± 0.023	0.391 ± 0.042	0.146 ± 0.036
Segmentation	Dice	0.979 ± 0.009	0.993 ± 0.004	0.932 ± 0.016	0.967 ± 0.008	0.899 ± 0.048
	Precision	0.979 ± 0.011	0.989 ± 0.009	0.920 ± 0.028	0.965 ± 0.008	0.863 ± 0.075
	Recall	0.986 ± 0.007	0.996 ± 0.002	0.945 ± 0.019	0.969 ± 0.011	0.942 ± 0.038

### Segmentation for Datasets With Noises

**FIGURE 7:**
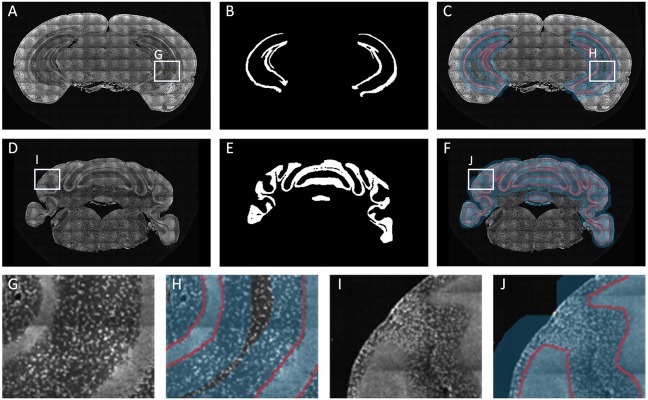
Segmentation effects for datasets with noises. **(A–C)** The coronal image, the predicted binarized image, the superposition of image, the localization masks, and the segmented lines for HIP-pg. **(D–F)** The same as A-C for CBX-gl. **(G–J)** Enlarged views of the areas in white boxes in **A**, **C**, **D**, and **F**.

For long-term continuous micro-optical imaging, it is easy to generate noises such as stripes and darkened corners through uneven illumination (Smith et al., 2015) of partial images in actual experiments. Noise makes the boundaries of brain regions more difficult to identify. In this section, we specially selected datasets with noises to verify the robustness of our method. We added some of these noisy samples into train set; then, after training, we predicted testing datasets. For HIP-pg and CBX-gl, Figure 7 shows the original images (A,D), the predicted binarized images (B,E) and the superpositions of images, the located Mask, and the predicted boundaries (C,F). The binarized images and the superposition images demonstrate that the localization and segmentation results on noisy images had the same good effect as on data without noise. Furthermore, Figures 7G–J show the details, illustrating that the segmented lines were well matched with the real boundaries, even in areas where the intensity difference was not obvious.

### Applicability of DeepBrainSeg for Other Types of Datasets

**FIGURE 8:**
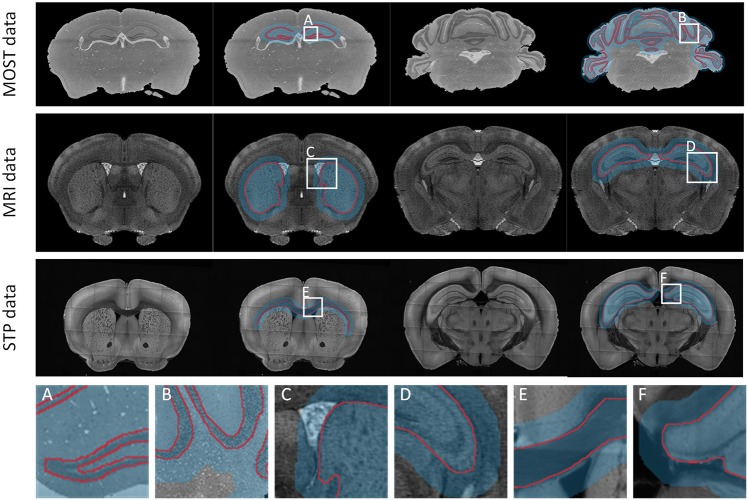
Applicability of DeepBrainSeg for other types of datasets. The first row shows the segmentation effects of HIP-pg and CBX-gl for MOST data. From left to right: the coronal image, the superposition of image, the localization masks, and the segmented lines for HIP-pg and CBX-gl. The second and third rows show CP and HIP for the MRI data, CC, and HIP for STP data, respectively. **(A–F)** Enlarged views of the areas in white boxes in the first three rows.

We validated the effectiveness, accuracy, and robustness of DeepBrainSeg using the BPS datasets. To further illustrate the applicability, we present the segmentation results for other types of data from: MOST, MRI, and STP systems. For datasets from these three imaging systems, we selected HIP-pg and CBX-gl, caudoputamen (CP) and hippocampus (HIP), corpus callosum (CC) and HIP, respectively, for segmentation. In Figure 8, the first three rows show both the original images and the superposition images with the located Mask and the segmented lines from each of the three systems. DeepBrainSeg was able to effectively segment the brain regions from multiple types of datasets. The fourth row shows enlarged images of the areas in white boxes (Figures 8A–F). The detail images reveal that the segmented lines closely matched the real boundaries, indicating the wide applicability of our method.

### Comparison With Other Methods

**FIGURE 9:**
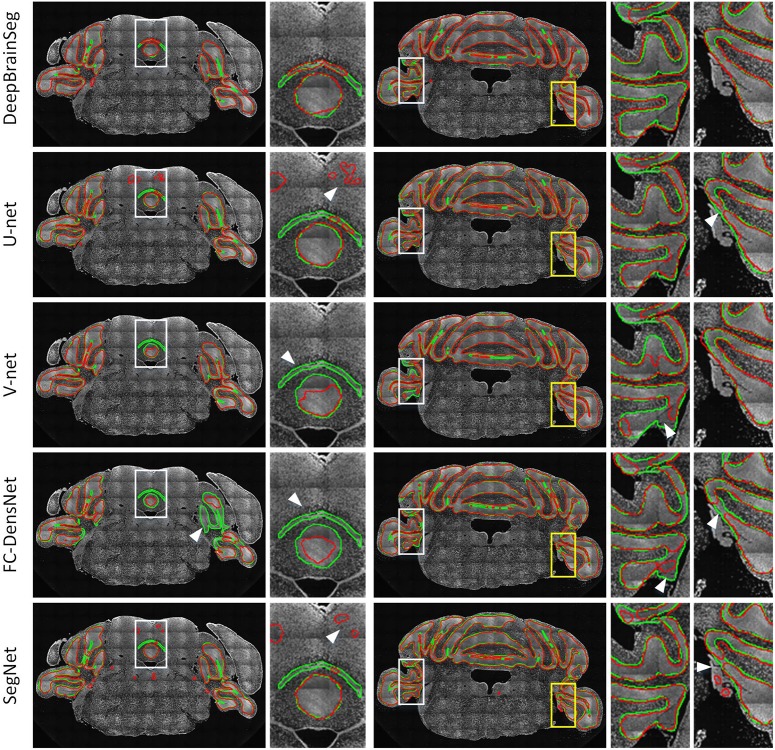
Comparison among DeepBrainSeg, U-net, V-net, FC-DenseNet, and SegNet. From top to bottom, the five rows show the segmentation effects of these methods, respectively. The first and third images in each row are superpositions of coronal images and the segmented lines: the green lines are the ground truth and the red lines are the automatically segmented lines. The second, fourth, and fifth images show enlarged views of the areas in front white boxes. White arrows show the inaccurate segmentations.

In this section, we compared DeepBrainSeg with other widely used methods including U-net, V-net, FC-DensNet, and Segnet. All methods were applied to BPS datasets with the same 60 training images and test images, and CBX-gl was selected as a representative structure with which to compare segmentation effects. The input images for DeepBrainSeg, U-net, V-net, and Segnet were full resolution of around 600 × 1000 pixels^2^, while for FC-DensNet, they are limited to 400 × 600 pixels^2^ due to the memory capacity of a GPU. Figure 9 shows the results of these methods from top to bottom. The green lines indicate the ground truth by manual segmentation, and the red lines are the automatically segmented lines. The second, fourth, and fifth columns are enlarged images of the white boxes in preceding columns. These results show that other methods achieved general segmentation effects: some over-segmentation and erroneous segmentation were present in the latter (marked by white arrows). In contrast, the segmented lines from DeepBrainSeg match more accurately with manual lines, and contain less erroneous segmentation. This indicates that DeepBrainSeg has a stronger segmentation ability for brain regions.

We also quantitatively evaluated the effects of the three methods in the test data. Table 3 shows the mean and standard deviation values of Dice, Precision, and Recall. Our proposed method achieves the highest values for the three parameters: 0.960, 0.953, and 0.968, respectively. In addition, we conducted statistical tests for evaluated values by conducting Wilcoxson test between DeepBrainSeg and others. The *P*-values displayed in Table 4, Dice, Precision, and Recall values of DeepBrainSeg are significantly different from all others (*P* < 0.05).

**TABLE 3:** Quantitative comparison among DeepBrainSeg and other methods (bold values are the highest).

	Dice	Precision	Recall
DeepBrainSeg	**0.960 ± 0.006**	**0.953 ± 0.009**	**0.968 ± 0.010**
U-net	0.929 ± 0.010	0.927 ± 0.019	0.931 ± 0.011
V-net	0.941 ± 0.007	0.929 ± 0.012	0.954 ± 0.016
FC-DensNet	0.919 ± 0.048	0.923 ± 0.014	0.922 ± 0.091
SegNet	0.911 ± 0.009	0.918 ± 0.018	0.904 ± 0.012

**TABLE 4:** *P*-values of Wilcoxson test among DeepBrainSeg and other methods.

	Dice	Precision	Recall
U-net	1.82e-04	7.69e-04	2.46e-04
V-net	3.30e-04	7.68e-04	3.12e-02
FC-DensNet	1.83e-04	1.00e-03	5.80e-03
SegNet	1.82e-04	2.46e-04	1.83e-04

### Performance Evaluation

**FIGURE 10:**
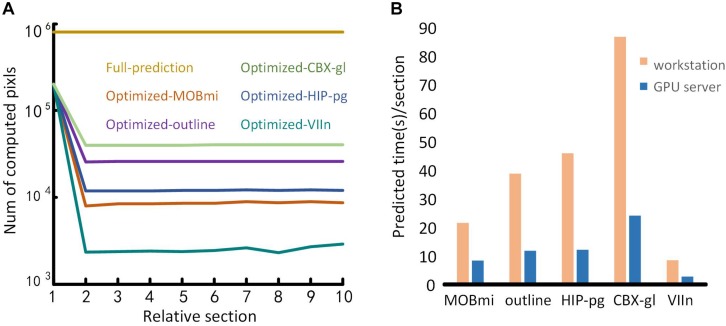
Performance testing. **(A)** Comparison of the time required for full-image predictions and optimization predictions. The abscissa represents a sequence of ten consecutive coronal images. The ordinate is the number of pixels to be calculated. Different color lines represent the calculation required for different brain regions using full-image predictions and the optimization predictions. **(B)** Performance of the proposed method on different computing platforms. The abscissa represents the five brain regions, and the ordinate is the average prediction time for each image. The orange and blue bars represent the performances of the workstation and the GPU server platform, respectively.

Benefiting from the optimization of the domain-condition constraint and prediction at intervals, our method significantly improved the computational efficiency. Ten consecutive coronal planes for five brain regions were selected to evaluate the number of pixels requiring computation before and after optimization, respectively. As shown in Figure 10A, when predicting each pixel in the entire image, the amount of calculation approaches 10^6^ for the full prediction. In contrast, using the optimization method, the first image requires three times less calculation. For subsequent images, only the pixels in the mask needed to be predicted, the amount of calculation decreased by one to three orders of magnitude according to the size of different brain regions.

To evaluate the performance of our method on different computing platforms, we tested five brain regions on a graphical workstation with a M6000 GPU and on a GPU server with four V100 GPUs. The prediction time of each section for the five brain regions on the platforms is shown in Figure 10B. The maximum runtime of one section was 90 s on the workstation. Furthermore, the time for that section decreased approximately threefold when executed on the GPU server platform.

## Discussion

In this study, following the principle of manual segmentation with multi-level features, we proposed DeepBrainSeg to solve the issue of brain region segmentation for micro-optical images based on a CNN. We used a dual-pathway CNN to learn local and contextual information at different scales, and provided global localization through image registration and domain-condition constraints. Our method can accurately segment multiple brain-wide regions, even for datasets with noises, and is widely applicable to various types of datasets. Moreover, it is superior to U-net, V-net, FC-DensNet, and Segnet in terms of accuracy.

We demonstrated the segmentation effects of our method on four different types of data. Furthermore, DeepBrainSeg can also be applied to solve segmentation problems in other fields for more types of data, such as computed tomography and electron microscopy. For other data, the patch size and network structure require adjustment according to the ratio of its resolution to 10 μm. Meanwhile, the potential regions for segmentation are not limited to the examples shown in this paper: the method is also suitable for other regions with characteristic differences to their surroundings. For brain region localization, DeepBrainSeg provides a location area that is consistent with the shape of the real brain region, rather than a regular shape like box. This irregular location area reduces the Redundancy to improve the localization accuracy and segmentation efficiency.

Nevertheless, our method still has some deficiencies. The training and prediction are implemented separately that target the characteristics of these different brain regions but introduce some complexity. Thus, finding one model that can segment multiple brain regions will be the subject of our future work. In addition, for efficiency, we processed datasets at an isotropic resolution of 10 μm. It is likely that a higher resolution could achieved by improving the algorithm and efficiency.

Research for brain space information involves collaborative analysis of various brain regions and datasets. Although many methods have been applied for brain segmentation, they are generally effective for only one type of data or a single brain region. Our intention is to provide neuroscientists with a consistently accurate segmentation framework that can be applied to multiple types of data and brain regions without requiring complex feature extraction or being subject to strict data-quality requirements. Users would only need to input the data into the method to quickly acquire satisfactory results. We believe that our method provides a powerful tool by which neuroscientists can explore the brain.

## Data Availability Statement

The image data and codes supporting the conclusions of this article will be made available by the authors, without undue reservation, to any qualified researcher.

## Ethics Statement

The animal study was reviewed and approved by the Institutional Animal Ethics Committee of Huazhong University of Science and Technology.

## Author Contributions

QL and HG conceived the project. CT and AL designed the method. CT, AL, and YG wrote the article. CT, ZF, HN, ZZ, and ZW processed the data sets. XL prepared the brain specimens. JY processed the brain-wide imaging.

## Conflict of Interest

The authors declare that the research was conducted in the absence of any commercial or financial relationships that could be construed as a potential conflict of interest.
